# Surgeon Volume and Surgeon Age in Relation to Proficiency Gain Curves for Prognosis Following Surgery for Esophageal Cancer

**DOI:** 10.1245/s10434-018-6869-8

**Published:** 2018-10-15

**Authors:** Eivind Gottlieb-Vedi, Hugh Mackenzie, Frans van Workum, Camiel Rosman, Pernilla Lagergren, Sheraz Markar, Jesper Lagergren

**Affiliations:** 1 Upper Gastrointestinal Surgery, Department of Molecular Medicine and Surgery, Karolinska Institutet, and Karolinska University Hospital, Stockholm, Sweden; 2 Department of Surgery and Cancer, Imperial College London, London, UK; 3 Department of Surgery, Radboudumc, Nijmegen, The Netherlands; 4 Surgical Care Science, Department of Molecular Medicine and Surgery, Karolinska Institutet, and Karolinska University Hospital, Stockholm, Sweden; 5 School of Cancer and Pharmaceutical Sciences, King’s College London, and Guy’s and St Thomas’ NHS Foundation Trust, London, UK

## Abstract

**Background:**

Surgery proficiency gain curves must be shortened to reduce patient harm during esophagectomy learning.

**Objective:**

This study aimed to test whether surgeon volume and surgeon age influenced the length of period of surgical proficiency gain.

**Methods:**

This population-based cohort study included 1384 patients with esophageal cancer who underwent esophagectomy by any of the 36 highest-volume surgeons in Sweden between 1987 and 2010, with follow-up until 2016. Annual surgeon volume was dichotomized by the median values into ‘higher-volume surgeons’ (≥ 4 cases per year) and ‘lower-volume surgeons’ (< 4 cases per year), and surgeon age at the start of practicing esophagectomies into ‘younger surgeons’ (aged < 45 years) and ‘older surgeons’ (aged ≥ 45 years). Proficiency gain curves were constructed using risk-adjusted cumulative sum analysis for 1- to 5-year mortality (main outcome) and secondary outcomes (presented below). The results were adjusted for all established prognostic factors.

**Results:**

For 1- to 5-year mortality, the change point was at 14 cases among ‘higher-volume surgeons’, while ‘lower-volume surgeons’ had a later change point at 31 cases. The corresponding change points were at 13 cases among ‘younger surgeons’ and at 48 cases among ‘older surgeons’. Similar patterns of differences in the proficiency gain curves were seen for the secondary outcomes of 30-day mortality and resection margin status (tumor involvement).

**Conclusion:**

Higher-volume- and younger surgeons seem to have a substantially shorter period of proficiency gain for long-term mortality and other outcomes following surgery for esophageal cancer. This indicates a value of intensified training of younger surgeons for these complex operations.

**Electronic supplementary material:**

The online version of this article (10.1245/s10434-018-6869-8) contains supplementary material, which is available to authorized users.

Esophageal cancer is the 6th most common cancer death globally,^1^ and the overall 5-year survival is < 20%.^2^,^3^ Curative treatment is resectional surgery (esophagectomy), usually in combination with neoadjuvant therapy.^4^ Proficiency gain for esophagectomies performed by individual surgeons influences both long- and short-term mortality.^5^ This needs to be solved to increase patient safety as surgeons gain proficiency in performing esophagectomy independently. However, no studies have investigated which factors affect surgical learning using clinical data. Other studies have established the critical prognostic role of individual surgeon volume (more than hospital volume),^6^ and how a certain surgeon age range (51–56 years) may optimize long-term survival for patients with esophageal cancer.^7^ The hypothesis of this study was that proficiency gain for reaching stable long-term mortality would require fewer case numbers if the surgeons have a higher annual volume of esophagectomies and are of lower age. Proposed mechanisms would be that learning is more effective if many operations are performed with greater intensity of practice, and that younger surgeons may be more receptive and more quickly learn to perform new and complex procedures. We also hypothesized that short-term mortality, rate of reoperations, rate of resection margin with cancer involvement, and lymph node harvest would be influenced by surgical proficiency gain. The aim of this study was to assess how annual surgeon volume and surgeon age influence the proficiency gain curves among surgeons performing resectional surgery for esophageal cancer.

## Methods

### Design

This was a population-based cohort study based on an updated version of a well-established Swedish cohort, which contains 98% of all surgically treated patients with esophageal cancer (adenocarcinoma or squamous cell carcinoma) in Sweden between 1987 and 2010, with follow-up until 31 May 2016.^5^ The study was approved by the Regional Ethical Review Board in Stockholm, Sweden.

### Source Cohort

The source cohort contained 1820 patients who underwent open esophagectomy for esophageal cancer during the study period. A detailed description is available elsewhere.^5^,^8^^–^10 In brief, patients diagnosed with esophageal cancer were identified from the *Swedish Cancer Registry*, which is 98% complete for esophageal cancer in Sweden.^11^ The *Swedish National Patient Registry* was used to identify patients who had undergone esophagectomy for cancer, for which the registry is 99.6% complete.^12^ The Patient Registry also provided complete and accurate information on medical comorbidities.^13^ Additionally, *medical records* for each patient were collected from Swedish hospitals: operation charts provided surgical approach, surgeon names, and annual surgeon volume, and data on tumor stage and histology were retrieved from histopathological records of the resected tumor specimens. The most common surgical procedure was a combined open abdominal and transthoracic approach (95%). Tumor stage was defined according to the TNM classification of the International Union Against Cancer.^14^*The Longitudinal Integration Database for Health Insurance and Labor Market Studies* provided information on the educational level of patients. Finally, the *Swedish Cause of Death Registry*, which is above 99% complete,^15^ provided date of death with follow-up until 31 May 2016.^7^ All data sources were cross-linked using the Swedish personal identity numbers, which are uniquely assigned to every Swedish resident at birth or immigration.^16^

### Study Cohort

For this study, birth dates of the surgeons were collected, which were retrieved from the *Swedish Registry of Licensed Health and Medical Care Personnel,* and used to calculate surgeon age. This registry was also used to collect the date of obtained specialist competence for each surgeon. The source cohort contained 139 surgeons, most of whom had small case series. The 36 surgeons with the largest case series (details given below) were included in this study. The other 103 surgeons were excluded due to insufficient cumulative volume to reach a plateau in the proficiency gain curves.

### Exposures

Exposures were annual surgeon volume and surgeon age. Annual surgeon volume was calculated by dividing the total number of operations for each surgeon by the number of years of practice. Years of practice was defined as date of specialization until retirement, assumed to be at 65 years of age, limited by the time period of the study. Surgeon age was defined as the age that each surgeon performed their first esophagectomy in the cohort. Using the median values as cut-offs, the 36 included surgeons were dichotomized into two groups of 18 surgeons for each exposure. Annual surgeon volume was categorized into ‘higher-volume surgeons’ (≥ 4 cases per year) and ‘lower-volume surgeons’ (< 4 cases per year), and surgeon age was categorized into ‘younger surgeons’ (< 45 years) and ‘older surgeons’ (≥ 45 years). Each surgeon had no experience prior to inclusion in the study because the primary surgeon had not conducted any esophagectomies for cancer before baseline.

To further contrast the effects of the exposures, the surgeons were also divided into tertiles, with 12 surgeons in each group for each exposure. In these analyses, annual surgeon volume was divided into groups with median annual volume of 2, 4, and 6 cases, and surgeon age was divided into groups of median ages of 38, 40, and 44 years.

### Outcomes

The main outcome was the number of cases of the proficiency gain curve to obtain a plateau in 1- to 5-year all-cause mortality rate following surgery. The four secondary outcomes were the case numbers of the proficiency gain curve needed for a stable 30-day all-cause mortality, reoperation rate (for any indication), resection margin status (R0 vs. R1), and total lymph node yield. A resection margin of R1 corresponded to microscopically visible tumor at the resection margin, and R0 represented no such tumor involvement.^17^

### Statistical Analysis

Follow-up was counted from the date of surgery until death, emigration, or end of study (31 May 2016), whichever occurred first. The operated patients were aligned in chronological order for each surgeon, from first to last. To identify the case numbers of the proficiency gain curves in the exposure groups, a combination of risk-adjusted cumulative sum (RA-CUSUM) and change-point analysis was performed. The RA-CUSUM curves were generated for the cumulative difference between the observed and the expected outcomes against patient number.^18^ The curves were plotted using the RA-CUSUM equation *S*_*i*_ = *S*_*i*−1_ + (Σ_–_–Σ_*R*_), where S_0_ = 0, S_*i*_ is the risk-adjusted cumulative sum at case number *i*, Σ_*i*_ is the sum of the observed outcome at case number *i*, and Σ_*R*_ is the sum of expected outcome at case number *i*. The curve goes upwards if the outcome is greater than expected, and downwards if less than expected. The expected probability for each case was calculated using multivariable logistic regression models. Seven potential confounding factors were adjusted for in the models: tumor stage (0–I, II, III, or IV),^10^ histological subtype (adenocarcinoma or squamous cell carcinoma),^19^ age of the patient (continuous),^10^ sex of the patient (male or female),^10^ use of neoadjuvant therapy (yes or no),^20^ Charlson comorbidity index score (0, 1, or ≥ 2, excluding esophageal cancer),^10^,^21^ and formal education of patients (< 10 years, 10–12 years, or > 12 years).^22^

The change point was defined as the patient number at which there was a sustained improvement in outcome. This was represented graphically on the RA-CUSUM curve as the maximal deflection of the curve, i.e. the point at which the outcome changed from worse to better than expected. The clinical significance of the change point was determined by comparing the outcomes before and after the identified change point. The results were also compared using the two-sided Mann–Whitney *U* test for continuous outcomes, and two-sided Chi square test for binomial outcomes, with a significance level of *p* < 0.05.

## Results

### Patients

Of 1820 patients in the source cohort, the 36 participating surgeons performed esophagectomies in 1384 patients (76.0%). Characteristics of these 1384 study patients are presented in Table 1. The median age was 66 years and the largest categories were male sex (74.2%), < 10 years of education (47.8%), no comorbidity [Charlson score 0] (56.6%), adenocarcinoma histology (54.8%), tumor stage II or III (68.2%), and no neoadjuvant therapy (66.9%).

**Table 1:** Characteristics of study participants having undergone esophagectomy for esophageal cancer in Sweden

Variable	No. of patients (%)
Total	1384 (100.0)
Age, years [median (interquartile range)]	66 (59–72)
Sex	
Male	1027 (74.2)
Female	357 (25.8)
Years of formal education	
< 10	662 (47.8)
10–12	494 (35.7)
> 12	189 (13.7)
Charlson comorbidity score	
0	784 (56.6)
1	289 (20.9)
≥ 2	311 (22.5)
Histological subtype	
Adenocarcinoma	759 (54.8)
Squamous cell carcinoma	622 (44.9)
Tumor stage	
0–I	336 (24.2)
II	507 (36.6)
III	437 (31.6)
IV	99 (7.2)
Neoadjuvant therapy	
Yes	458 (33.1)
No	926 (66.9)

### Surgeons

Among the 36 participating surgeons, ‘higher-volume surgeons’ performed a median annual volume of five cases (interquartile range [IQR] 4–6), whereas ‘lower-volume surgeons’ performed a median annual volume of two cases (IQR 1–3). ‘Younger surgeons’ had a median age of 40 years (IQR 37–42 years) at the first performed case compared with 47 years (IQR 46–53) for ‘older surgeons’.

### Annual Surgeon Volume and Proficiency Gain Curves

#### Mortality Within 1–5 Years of Surgery

**Fig. 1:**
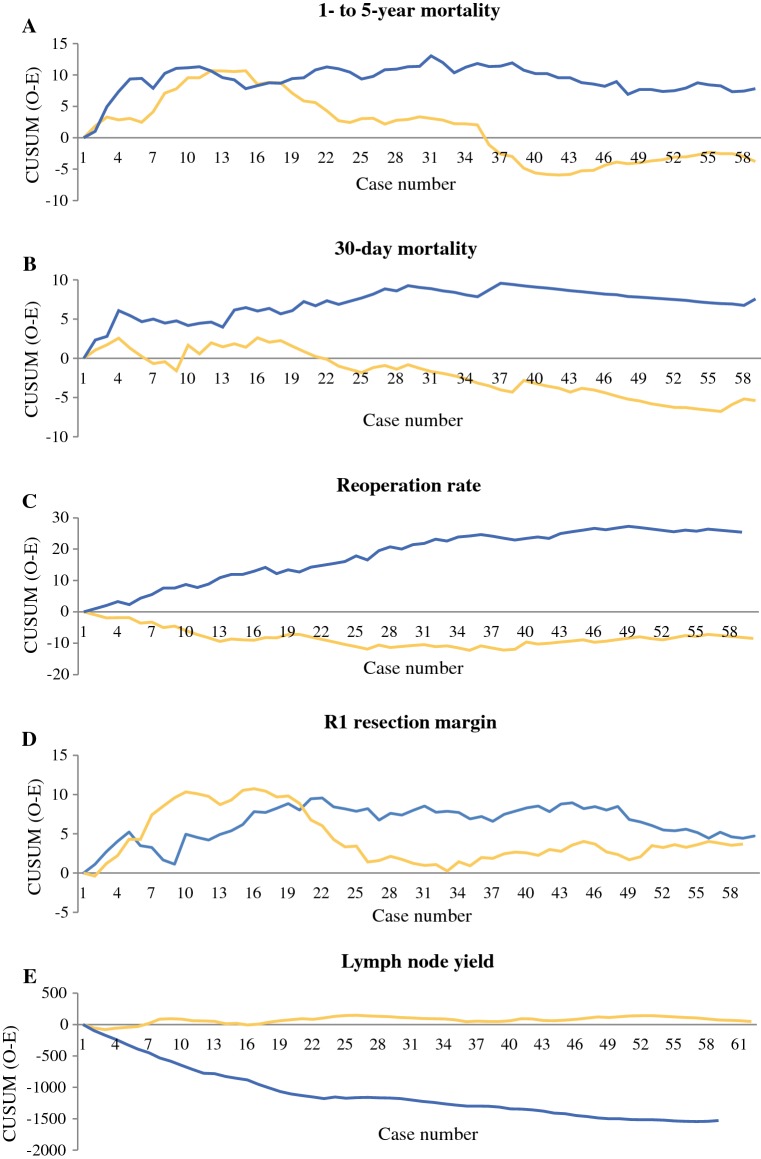
Proficiency gain curves in relation to ‘higher-volume surgeons’ (yellow lines) and ‘lower-volume surgeons’ (blue lines) in relation to **a** 1- to 5-year mortality, showing change point for ‘higher-volume surgeons’ at 14 cases and ‘lower-volume surgeons’ at 31 cases; **b** 30-day mortality, showing change point for ‘higher-volume surgeons’ at 16 cases and ‘lower-volume surgeons’ at 37 cases; **c** reoperation rate, showing no change points; **d** R1 resection margin, showing change point for ‘higher-volume surgeons’ at 16 cases and ‘lower-volume surgeons’ at 22 cases; **e** lymph node yield, showing no change points. *CUSUM* cumulative sum

For ‘higher-volume surgeons’, the change point for 1- to 5-year mortality was at 14 cases (Fig. 1a), where the mortality decreased from 67.2% to 57.1% (*p* = 0.049) [Table 2]. For ‘lower-volume surgeons’, the change point was at 31 cases (Fig. 1a), where the mortality decreased from 67.8 to 64.9% (*p* = 0.875) [Table 2].

**Table 2:** Outcomes among 1384 patients having undergone esophagectomy for esophageal cancer before and after change points in proficiency gain curves, comparing 18 ‘higher-volume surgeons’ with 18 ‘lower-volume surgeons’

Outcome	Annual volume	Proficiency gain curve change point (*n*)	Percentage with outcome (number/total number)		
			Before change point	After change point	Change-point *p* value
1- to 5-year mortality	Higher	14	67.2% (82/122)	57.1% (216/378)	0.049
	Lower	31	67.8% (187/276)	64.9% (50/77)	0.875
30-day mortality	Higher	16	4.5% (10/221)	2.5% (15/589)	0.047
	Lower	37	5.3% (26/490)	3.6% (3/84)	0.502
Reoperation rate	Higher	No	NA	NA	NA
	Lower	No	NA	NA	NA
R1 resection margin	Higher	16	20.9% (49/235)	13.0% (62/476)	0.027
	Lower	22	21.5% (50/233)	16.0% (40/252)	0.081
Lymph node yield	Higher	No	NA	NA	NA
	Lower	No	NA	NA	NA

*NA* not applicable

#### 30-Day Mortality

For ‘higher-volume surgeons’, the change point for 30-day mortality was at 16 cases (Fig. 1b), where the mortality decreased from 4.5 to 2.5% (*p* = 0.047) [Table 2]. For ‘lower-volume surgeons’, the corresponding change point was at 37 cases (Fig. 1b), where the mortality decreased from 5.3 to 3.6% (*p* = 0.502) [Table 2].

#### Reoperation Rate

The reoperation rate was 7.7% for ‘higher-volume surgeons’ compared with 15.2% for ‘lower-volume surgeons’ (*p* < 0.001), but there were no identifiable change points for either curve (Fig. 1c).

#### Resection Margin Status

For ‘higher-volume surgeons’, the change point for the R1 resection margin status was at 16 cases (Fig. 1d), where it decreased from 20.9 to 13.0% (*p* = 0.027) [Table 2]. For ‘lower-volume surgeons’, the corresponding change point was at 22 cases (Fig. 1d), where it decreased from 21.5 to 16.0% (*p* = 0.081) [Table 2].

#### Lymph Node Yield

The number of lymph nodes removed and examined was higher for ‘higher-volume surgeons’ (median 11 [IQR 5–20]) compared with ‘lower-volume surgeons’ (median 5 [IQR 3–11]) (*p* < 0.001), but no change points were present in the graphs (Fig. 1e).

### Surgeon Age and Proficiency Gain Curves

#### Mortality Within 1–5 Years of Surgery

**Fig. 2:**
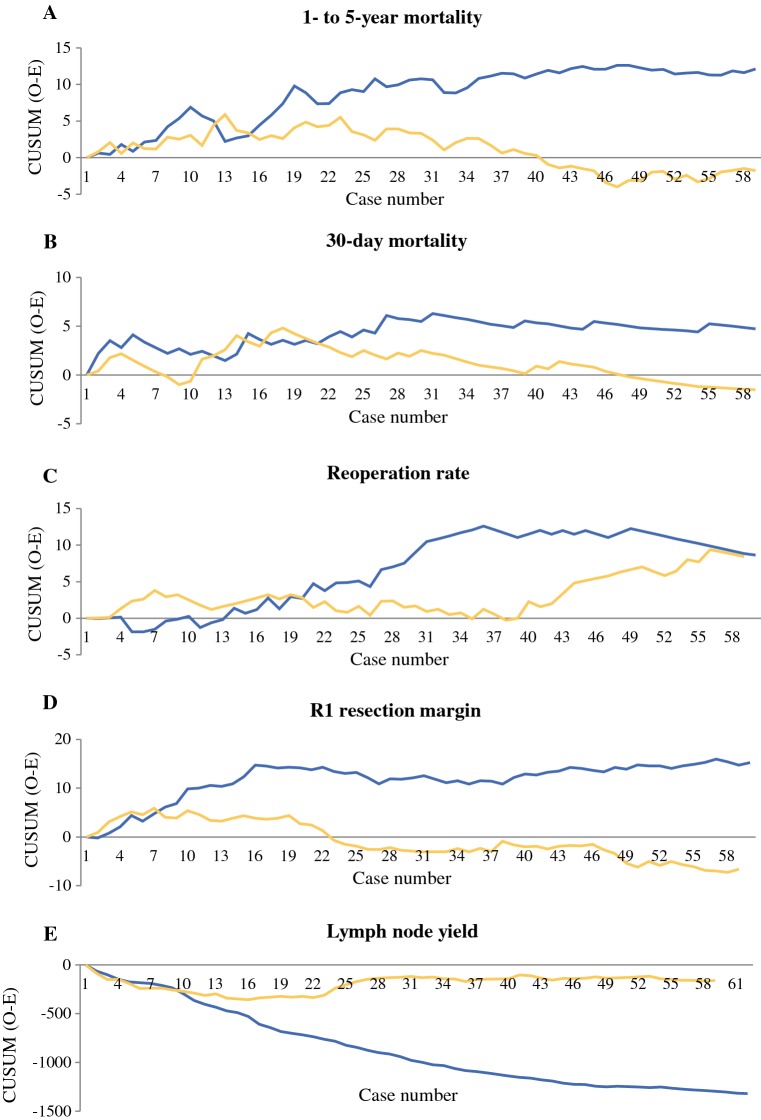
Proficiency gain curves in relation to ‘younger surgeons’ (yellow lines) and ‘*older surgeons*’ (blue lines) in relation to **a** 1- to 5-year mortality, showing change point for ‘younger surgeons’ at 13 cases and ‘older surgeons’ at 48 cases; **b** 30-day mortality, showing change point for ‘younger surgeons’ at 18 cases and ‘older surgeons’ at 31 cases; **c** reoperation rate, showing no change point for ‘younger surgeons’ and for ‘older surgeons’ at 36 cases; **d** R1 resection margin, showing change point for ‘younger surgeons’ at 7 cases and ‘older surgeons’ at 16 and 56 cases; **e** lymph node yield, showing no change points. *CUSUM* cumulative sum

For ‘younger surgeons’, the change point for 1- to 5-year mortality was at 13 cases (Fig. 2a), where the mortality decreased from 63.4 to 56.9% (*p* = 0.191) [Table 3]. For ‘older surgeons’, the corresponding change point was at 48 cases (Fig. 2a), where the mortality decreased from 65.5 to 52.2% (*p* = 0.201) [Table 3].

**Table 3:** Outcomes among 1384 patients having undergone esophagectomy for esophageal cancer before and after change points in proficiency gain curves, comparing 18 ‘younger surgeons’ with 18 ‘older surgeons’

Outcome	Age group	Proficiency gain curve change point (*n*)	Percentage with outcome (number/total number)		
			Before change point	After change point	Change-point *p* value
1- to 5-year mortality	Younger	13	63.4% (78/123)	56.9% (257/452)	0.191
	Older	48	65.5% (167/255)	52.2% (12/23)	0.201
30-day mortality	Younger	18	5.2% (15/288)	2.4% (15/625)	0.027
	Older	31	5.7% (21/366)	2.9% (3/105)	0.237
Reoperation rate	Younger	No	NA	NA	NA
	Older	No	NA	NA	NA
R1 resection margin	Younger	7	18.8% (21/112)	14.9% (103/693)	0.290
	Older (1)^a^	16	25.0% (49/196)	17.4% (34/195)	0.067
	Older (2)^a^	56	21.3% (81/380)	18.2% (2/11)	0.802
Lymph node yield	Younger	No	NA	NA	NA
	Older	No	NA	NA	NA

*NA* not applicable

^a^Two change points

#### 30-Day Mortality

For ‘younger surgeons’, the change point for 30-day mortality was at 18 cases (Fig. 2b), where the mortality decreased from 5.2 to 2.4% (*p* = 0.027) [Table 3]. For ‘older surgeons’, the corresponding change point was at 31 cases (Fig. 2b), where the mortality decreased from 5.7 to 2.9% (*p* = 0.237) [Table 3].

#### Reoperation Rate

There was no obvious change point for ‘younger surgeons’ (Fig. 2c). ‘Older surgeons’ had a change point for reoperation rate at 36 cases, where it decreased from 14.4 to 9.8% (*p* = 0.265). However, ‘younger surgeons’ had a lower reoperation rate (9.3%) than ‘older surgeons’ (13.6%) [*p* = 0.015].

#### Resection Margin Status

For ‘younger surgeons’, the change point for the R1 resection margin was at 7 cases (Fig. 2d), where it decreased from 18.8 to 14.9% (*p* = 0.290) [Table 3]. For ‘older surgeons’, two change points were present, one at 16 cases and the other at 56 cases (Fig. 2d), where it decreased from 25.0 to 17.4% (*p* = 0.067) and 21.3–18.2% (*p* = 0.802), respectively (Table 3).

#### Lymph Node Yield

Regarding lymph node yield, no change points were present for ‘younger’ or ‘older’ surgeons (Fig. 2e). However, the number of lymph nodes removed and examined was higher for ‘younger surgeons’ (median 11 [IQR 5–20]) compared with ‘older surgeons’ (median 5 [IQR 3–9]) [*p* < 0.001].

### Proficiency Gain Curves of Volume and Age Tertiles

The RA-CUSUM curves of the tertiles consistently demonstrated that ‘higher-volume surgeons’ and ‘younger surgeons’ had earlier change points than their respective counterparts, with the middle tertile change points in between (electronic supplementary material). Change-point analyses were not repeated due to few numbers in each tertile.

## Discussion

This study indicates that higher-volume surgeons and younger surgeons have shorter proficiency gain curves for long-term survival following surgery for esophageal cancer than lower-volume surgeons and older surgeons, respectively. Similar improvements were seen in the proficiency gain curves for 30-day mortality and resection margin status. The reoperation rates were lower and lymph node yield was higher for higher-volume surgeons and younger surgeons compared with their respective comparison groups, but no change points were identified.

Advantages of the study include the population-based design with a high (98%) participation rate and long and complete follow-up of all patients, and the usage of accurate and complete patient and surgeon data on exposures, outcomes, and confounders. Limitations include the retrospective design that possibly prevents collection and examination of all potential confounding variables. However, the data assessment was comprehensive and included the medical records of all patients. Although it is possible that the defined case 1 may not have been the first case performed by the surgeons, the long study period (from 1987 onwards) meant that this might have occurred only in a minority of cases. The cut-offs for defining ‘higher-volume surgeons’ and ‘younger surgeons’ were based on the median values and may not be representative of other datasets, limiting the external validity of the results. As in any observational study, there might have been unknown selection bias and confounding, including selection of cases to certain experienced surgeons, as well as changes that occurred during the long study period. However, the results were adjusted for all established prognostic factors,^10^,^19^,^20^,^22^ which is a major strength. The vast majority of the operations within the Swedish population cohort was performed by the 36 included surgeons. Nevertheless, the total number of surgeons analyzed was limited, which reduced the statistical power, and might explain why some change points were not statistically significant. However, the change points were evident from the RA-CUSUM plots and followed a distinct and consistent pattern for surgeon volume and starting surgeon age on the case numbers of the proficiency gain curves. The difference in annual volume between the higher- and lower-volume surgeons was small, but the study detected substantial differences in case numbers at which the surgeons reached a stable plateau for most outcomes. This is also true regarding surgeon age; however, this study does not demonstrate the optimal annual volume or surgeon age. It may be that even younger and higher-volume surgeons learn faster than shown by this study. The training period or residency was not captured within the present study; however, none of the surgeons had any past primary surgeon experiences of esophagectomies prior to being included in the study. Similarly, the age of each surgeon was defined at case 1, meaning that the older surgeons in the study are only more senior by age, but not by experience. Therefore, it is unlikely that there is bias due to the difficulty of cases. In addition, the curves were risk-adjusted according to the case demographics available, which were comprehensive for case complexity. The study period was from 1987 to 2010, which preceded centralization of esophageal cancer surgical services in Sweden. However, in the current era, surgeons more commonly operate in teams, and the proficiency gain curves in future may need to be modeled by teams rather than individual surgeons.

The role of proficiency gain curves in the long-term prognosis following esophagectomy has been examined based on an earlier version of the cohort used for the present study.^5^ The study found that prognosis was better after the proficiency gain curve had been completed. This indicates that patient safety is compromised during surgical proficiency gain and that patients can benefit from more efficient surgical learning. Previous studies have shown improved long-term survival for surgeons with higher volume for esophagectomies.^8^,^23^,^24^ Importantly, in the same cohort we have clearly shown greater prognostic importance of surgeon volume over hospital volume, and thus the present study focused on the influence of surgeon volume on the proficiency gain curve.^8^ A study on surgeon age showed that both short- and long-term survival after esophagectomies was better for surgeons aged 52–56 years.^7^ This is possibly explained by a trade-off between accumulated competence and decreased technical skills and concentration with increased age.

To the best of our knowledge, this is the first study investigating how proficiency gain curves are influenced by annual surgeon volume and surgeon age. A possible explanation for the ‘surgeon volume effect’ is that a higher annual volume during a shorter time period facilitates a more effective acquirement of new skills. A possible explanation for the results regarding surgeon age is that younger surgeons may be more responsive to obtaining new skills, both intellectually and practically, relative to their older counterparts. As the age of each surgeon was defined at their case 1, referral of more complicated cases to older surgeons cannot explain the results.

The findings of this study suggest the need to establish systematic training programs for selected surgeons to conduct esophagectomies in order to minimize learning-associated mortality. The results suggest that cases should be converged towards fewer and possibly younger surgeons, providing them with adequate volume within a shorter period of time. Collaboration between hospitals, or centralization of esophagectomies to high-volume hospitals, may be needed to provide a sufficient procedural volume during a limited period of training. Although the present study only included open esophagectomies, it is possible that the results can also be applied to minimally invasive esophagectomies, but future studies are needed to examine this more closely.

## Conclusion

This comprehensive nationwide Swedish cohort study indicates that higher annual surgeon volume and younger surgeon age shortens proficiency gain curves of open esophagectomies for esophageal cancer. This indicates the need for well-organized and intense training of surgeons in esophageal cancer surgery.

## Electronic Supplementary material

Below is the link to the electronic supplementary material.

Supplementary material 1 (DOCX 306 kb)
