# Supplementary material for: Surgeon Volume and Surgeon Age in Relation to Proficiency Gain Curves for Prognosis Following Surgery for Esophageal Cancer
Source: Ann Surg Oncol. 2018 Oct 15;26(2):497–505. doi: 10.1245/s10434-018-6869-8 (PMC6341157; doi:10.1245/s10434-018-6869-8)
Supplement: Supplementary file 1 — Supplementary material 1 (DOCX 306 kb) [file 10434_2018_6869_MOESM1_ESM.docx]

**Supplementary figure 1. Proficiency gain curves in relation to “higher-volume surgeons” (red lines), “medium-volume surgeons” (green lines) and “lower-volume surgeons” (blue lines) in relation to:**

**(A) 1 to 5-year mortality.**

**(B) 30-day mortality.**

**(C) Reoperation rate.**

**(D) R1 resection margin.**

**(E) Lymph node yield.**


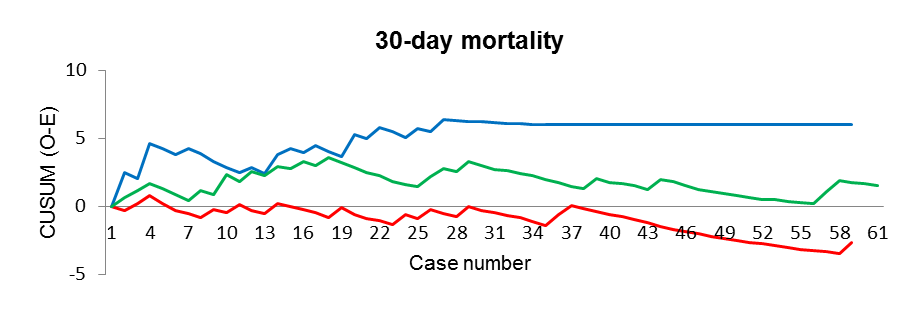

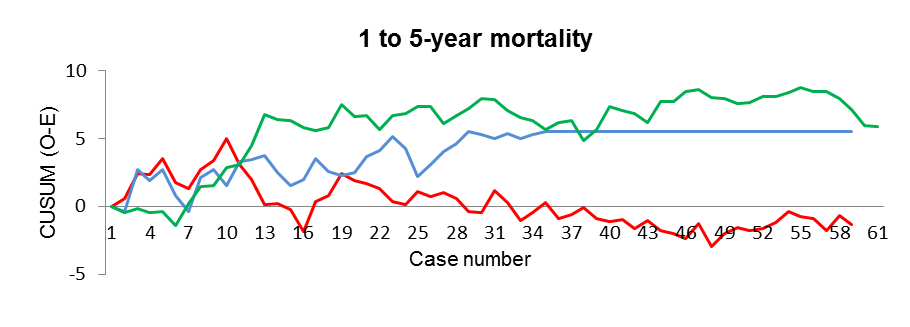

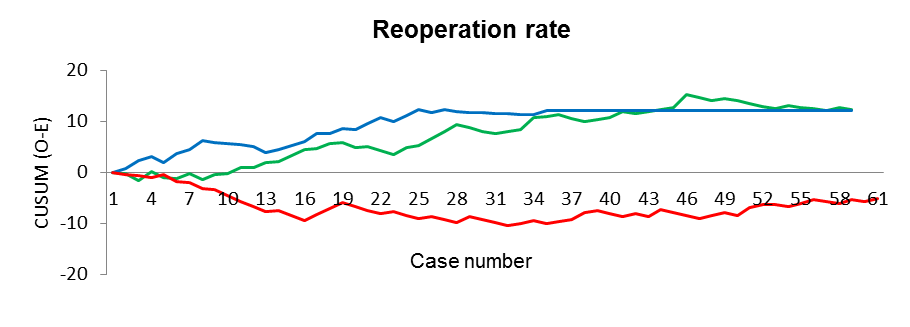

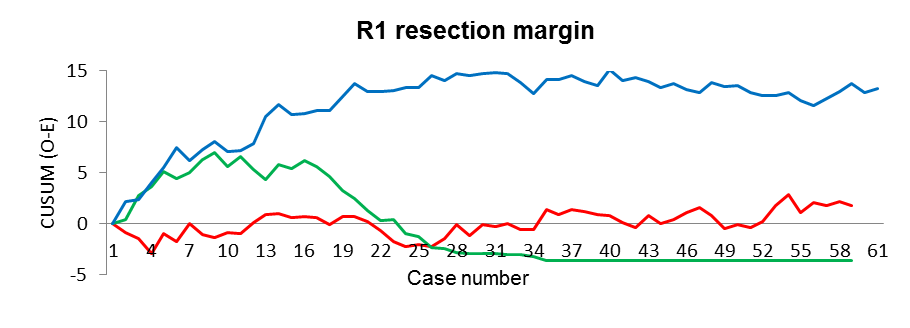

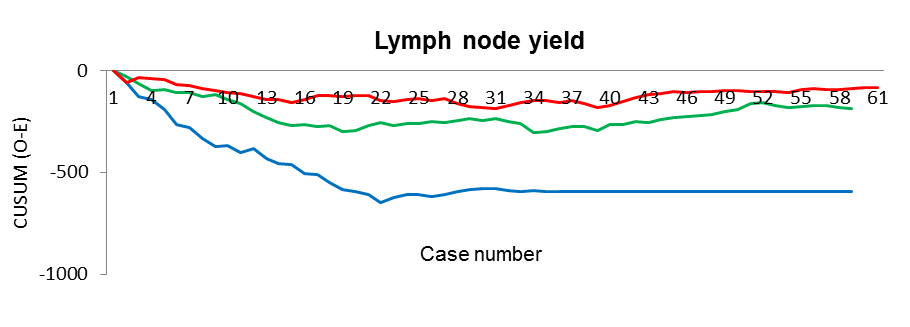


**A**

**B**

**C**

**D**

**E**

**Supplementary figure 2. Proficiency gain curves in relation to “younger surgeons” (red lines), “medium-aged surgeons” (green lines) and “older surgeons” (blue lines) in relation to:**

**(A) 1 to 5-year mortality.**

**(B) 30-day mortality.**

**(C) Reoperation rate.**

**(D) R1 resection margin.**

**(E) Lymph node yield.**


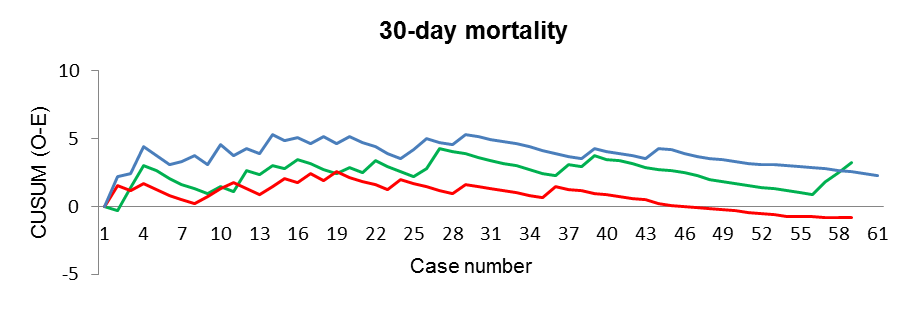

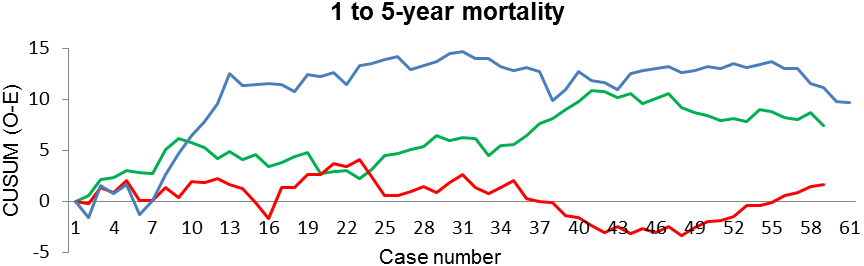

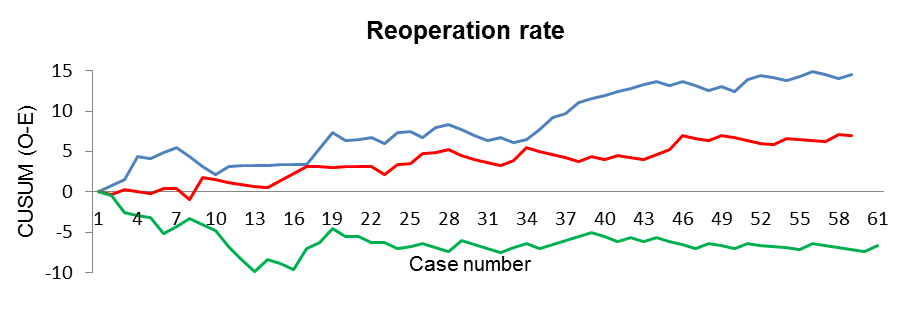

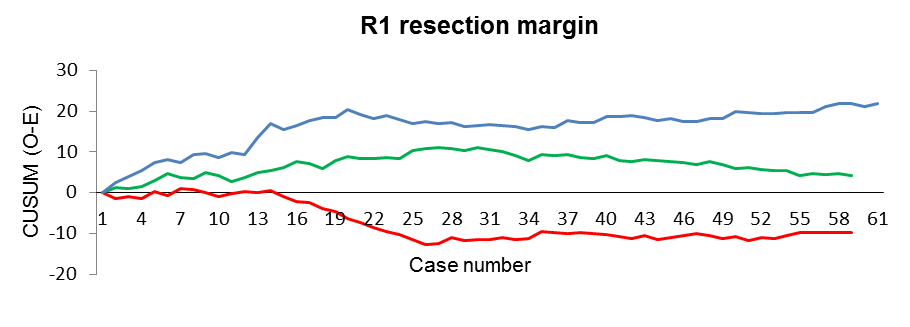

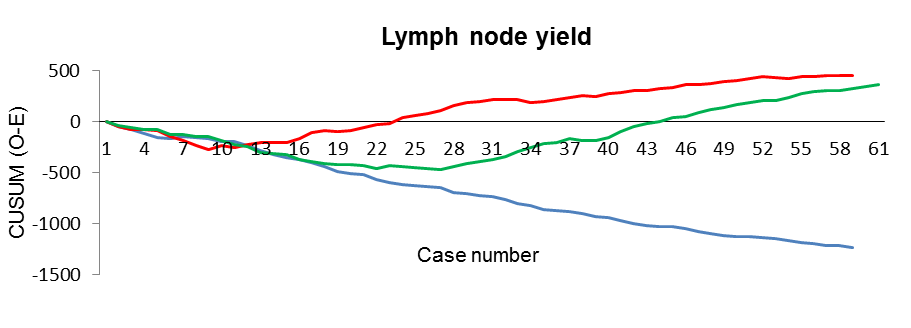


**A**

**B**

**C**

**D**

**E**
